# Supplementary material for: Causal Relationship between Aging and Anorexia Nervosa: A White-Matter-Microstructure-Mediated Mendelian Randomization Analysis
Source: Biomedicines. 2024 Aug 16;12(8):1874. doi: 10.3390/biomedicines12081874 (PMC11351342; doi:10.3390/biomedicines12081874)
Supplement: Supplementary file 1 [file biomedicines-12-01874-s001.zip › Supplementary Figure/Figure S4/Singe SNP plot of MR analysis for TL to ubm-b-1955.pdf]

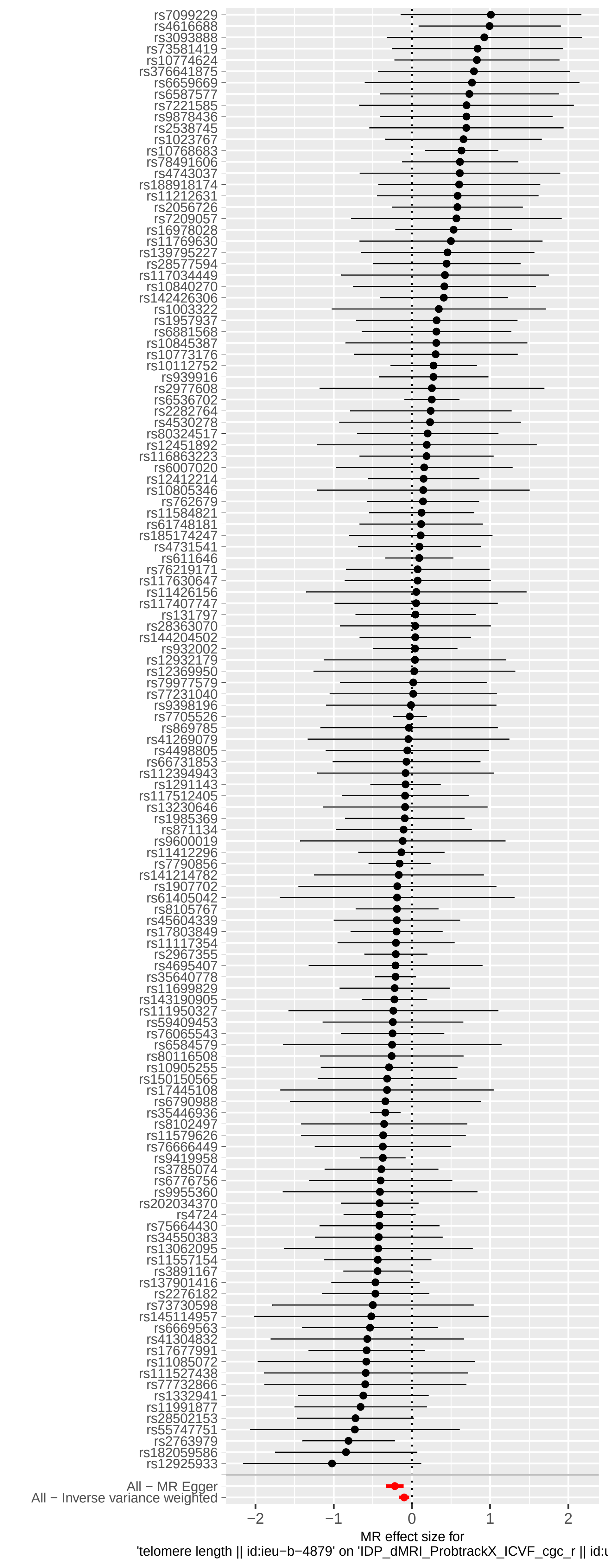
rs7099229
rs4616688
rs3093888
rs73581419
rs10774624
rs376641875
rs6659669
rs6587577
rs7221585
rs9878436
rs2538745
rs1023767
rs10768683
rs78491606
rs4743037
rs188918174
rs11212631
rs2056726
rs7209057
rs16978028
rs11769630
rs139795227
rs28577594
rs117034449
rs10840270
rs142426306
rs1003322
rs1957937
rs6881568
rs10845387
rs10773176
rs10112752
rs939916
rs2977608
rs6536702
rs2282764
rs4530278
rs80324517
rs12451892
rs116863223
rs6007020
rs12412214
rs10805346
rs762679
rs11584821
rs61748181
rs185174247
rs4731541
rs611646
rs76219171
rs117630647
rs11426156
rs117407747
rs131797
rs28363070
rs144204502
rs932002
rs12932179
rs12369950
rs79977579
rs77231040
rs9398196
rs7705526
rs869785
rs41269079
rs4498805
rs66731853
rs112394943
rs1291143
rs117512405
rs13230646
rs1985369
rs871134
rs9600019
rs11412296
rs7790856
rs141214782
rs1907702
rs61405042
rs8105767
rs45604339
rs17803849
rs11117354
rs2967355
rs4695407
rs35640778
rs11699829
rs143190905
rs111950327
rs59409453
rs76065543
rs6584579
rs80116508
rs10905255
rs150150565
rs17445108
rs6790988
rs35446936
rs8102497
rs11579626
rs76666449
rs9419958
rs3785074
rs6776756
rs9955360
rs202034370
rs4724
rs75664430
rs34550383
rs13062095
rs11557154
rs3891167
rs137901416
rs2276182
rs73730598
rs145114957
rs6669563
rs41304832
rs17677991
rs11085072
rs111527438
rs77732866
rs1332941
rs11991877
rs28502153
rs55747751
rs2763979
rs182059586
rs12925933
All − MR Egger
All − Inverse variance weighted
−2
−1
0
1
2
MR effect size for
'telomere length || id:ieu−b−4879' on 'IDP_dMRI_ProbtrackX_ICVF_cgc_r || id:u
